# Trends in management and outcomes of pregnant women living with HIV between 2008–2013 and 2014–2019: A retrospective cohort study

**DOI:** 10.3389/fmed.2022.970175

**Published:** 2022-10-20

**Authors:** Olivia Brandon, Sujay Chakravarti, Joris Hemelaar

**Affiliations:** ^1^National Perinatal Epidemiology Unit, Oxford Population Health, University of Oxford, Oxford, United Kingdom; ^2^Department of Obstetrics, John Radcliffe Hospital, Oxford University Hospitals National Health Service (NHS) Trust, Oxford, United Kingdom

**Keywords:** HIV, antiretroviral therapy, pregnancy, perinatal outcome, integrase inhibitor

## Abstract

**Background:**

Despite major advances in the care of pregnant women living with HIV (WLHIV), they remain at increased risk of adverse pregnancy outcomes. This study assesses recent developments in management and outcomes of pregnant WLHIV at a tertiary obstetric unit in the United Kingdom.

**Methods:**

We conducted a retrospective cohort study of WLHIV delivering at the John Radcliffe Hospital, Oxford, during 2008–2019. Detailed data was collected for maternal, virological, obstetric, and perinatal characteristics. To determine changes over time, data from the periods 2008–13 and 2014–19 were compared.

**Results:**

We identified 116 pregnancies in 94 WLHIV. Between 2008–2013 and 2014–2019, the rate of preconception HIV diagnosis increased from 73 to 90% (*p* = 0.021) and the proportion of WLHIV on combination ART (cART) at conception increased from 54 to 84% (*p* = 0.001). The median gestation at which cART was initiated antenatally decreased from 22+1 to 17+1 weeks (*p* = 0.003). In 2014-2019, 41% of WLHIV received non-nucleoside reverse transcriptase inhibitor-based cART, 37% protease inhibitor-based cART, and 22% of cART regimens contained an integrase inhibitor. The proportion of WLHIV with a viral load <50 copies/mL at delivery rose from 87 to 94% (*p* = 0.235). Sixty-six percent of WLHIV delivered by Cesarean section, with a significant decrease over time in the rate of both planned (62–39%, *p* = 0.016) and actual (49–31%, *p* = 0.044) elective Cesarean. Perinatal outcomes included one case of perinatal HIV transmission (0.86%), 11% preterm birth, 15% small-for-gestational-age, and 2% stillbirth. There was an association between a viral load >50 copies/mL at delivery and preterm delivery (*p* = 0.0004).

**Conclusion:**

Virological, obstetric, and perinatal outcomes of WLHIV improved during the study period. Implementation of national guidance has led to an increase in preconception diagnosis and treatment, earlier initiation of antenatal treatment, a reduction in the number of women with a detectable viral load at delivery, and an increase in vaginal deliveries.

## Introduction

In 2015, the prevalence of HIV in pregnant women in the United Kingdom (UK) was 0.31%, with rates of around 0.6% in London (1). Over the past 30 years, major advances have been made in improving the health of pregnant women living with HIV (WLHIV), as well as reducing the vertical transmission of HIV. The implementation of universal antenatal screening for HIV from 1999 onwards has been key in the early detection of HIV in pregnant women in the UK. In 2018, antenatal screening coverage in the UK reached 99.6% (2). Other measures crucial to the reduction of vertical transmission of HIV included treatment with intrapartum zidovudine in WLHIV with a detectable viral load, exclusive formula feeding, and neonatal ART. Although elective cesarean was initially recommended in all WLHIV, it is currently only considered in those with a detectable viral load at 36 weeks gestation (3). Antenatal zidovudine monotherapy was widely implemented from 1994 onwards, after it was demonstrated to reduce the risk of vertical transmission of HIV (4). Antenatal combination antiretroviral therapy (cART) was subsequently shown to be superior in reducing vertical HIV transmission and replaced the use of zidovudine monotherapy (5). From 2015, the World Health Organization (WHO) recommend that all people living with HIV should initiate cART as soon as possible after diagnosis, including pregnant WLHIV (6). As a result, global vertical HIV transmission rates fell by 41% between 2010 and 2018 (7). Likewise, rates of vertical HIV transmission in the UK have fallen from 25.6% in 1993, to 2.1% in 2000–2001, to 0.28% in 2015–2016 (8–11).

Untreated maternal HIV infection is associated with an increased risk of adverse perinatal outcomes, including preterm birth, low birth weight, small for gestational age (SGA), and stillbirth (12). Maternal cART improves maternal health and reduces vertical HIV transmission, but the impact on adverse perinatal outcomes has been controversial and may vary by ART regimen and timing of ART initiation (13–21). Current best practice for the care of pregnant WLHIV in the UK is guided by the British HIV Association (BHIVA) guidelines (3). Implementation of these guidelines is key to improving pregnancy outcomes for WLHIV, but detailed and up-to-date information on the management and outcomes of pregnant WLHIV in the UK is sparse. We conducted a retrospective cohort study to assess maternal characteristics, clinical management, and virological, obstetric and perinatal outcomes among WLHIV in a tertiary hospital in the UK in the period 2008–2019.

## Materials and methods

We conducted a retrospective cohort study of pregnant WLHIV who delivered at the John Radcliffe Hospital, Oxford, UK between January 2008 and October 2019. Eligible patients were identified using the electronic hospital records database.

Data were extracted from electronic records, paper notes, and National Surveillance of HIV in Pregnancy and Childhood (NSHPC) reporting forms. The data collected included 136 items on maternal demographics, sexual health, contraception, social history, obstetric history, HIV laboratory investigations, antiretroviral therapy, antenatal care, delivery, and neonatal outcomes. Gestational age was based on the first trimester dating scan, except for those who booked late, who instead had dating scans in the second or third trimester. One patient had a concealed pregnancy and gestational age was estimated by neonatal assessment. Preterm birth was defined as delivery before 37^+0^ weeks. Gestation and sex-adjusted birthweight centiles were calculated using the Intergrowth-21st neonatal size calculator (22). Small for gestational age (SGA) was defined as < 10th centile and large for gestational age (LGA) as >90th centile. All data were anonymized and stored in password protected electronic files in Microsoft Excel. The study was registered and approved by the institutional governance department.

In order to analyze changes in patient characteristics, clinical management, and virological, obstetric and perinatal outcomes over time, the study cohort was split into two groups. Given that guidelines on the management of HIV in pregnancy have changed multiple times over the study period, we chose to divide our data evenly based on years of delivery (2008–2013 and 2014–2019).

In WLHIV who had more than one pregnancy in the study period, each pregnancy was treated as a separate case. Differences in frequencies and medians between the two time periods were compared using the Chi-squared test, Fisher's exact test, or the Mann-Whitney *U*-test, as appropriate. The associations of key aspects in HIV management with perinatal outcomes were assessed using the same statistical tests. All statistical analyses were performed in Prism (version 9.1.0, GraphPad Software, San Diego, California, USA). *p* < 0.05 was considered statistically significant.

## Results

During the period 2008–2019, we identified 129 pregnancies among WLHIV who delivered at the John Radcliffe Hospital, Oxford. Thirteen WLHIV were excluded due to missing medical notes. The remaining 116 pregnancies occurred among 94 different WLHIV, including 16 WLHIV who had two pregnancies in the study period, and three who had three pregnancies. Sixty-seven WLHIV delivered in the period 2008–2013 and 49 in 2014–2019.

Between 2008–2013 and 2014–2019, the rate of preconception HIV diagnosis increased from 72 to 90% (*p* = 0.021; Table 1). Conversely, HIV diagnosis during pregnancy, as part of the antenatal screening program, decreased from 26 to 10% (*p* = 0.033). Antenatal HIV diagnosis also tended to be made earlier in pregnancy, with median gestation 15^+0^ weeks in 2008–2013 and 11^+5^ weeks in 2014–2019 (*p* = 0.140). Overall, 30% of WLHIV diagnosed antenatally had a CD4 count < 350 cells/mm^3^, and 26% had a CD4 count <200 cells/mm^3^. Two percent of WLHIV had symptomatic HIV infection, and 7% were diagnosed with acquired immunodeficiency syndrome (AIDS; Table 1).

**Table 1:** Maternal characteristics.

**Characteristic^a^**	**Whole cohort (2008–19)^b^**	**2008–13^b^**	**2014–19^b^**	***P*-value^e^**
Ethnic origin (*n* = 115, 67, 49)				
Black African	92 (80)	55 (83)	37 (76)	0.300
Black Caribbean	1 (1)	0 (0)	1 (2)	0.426
White	11 (10)	6 (9)	5 (10)	0.841
Other	11 (10)	5 (8)	6 (12)	0.400
Born in the UK (*n* = 111, 62, 49)	9 (8)	4 (6)	5 (10)	0.505
Age at conception, y (*n* = 115, 66, 49)	32 (29–37)	31 (29–36)	34 (31–37)	0.013
Age at HIV diagnosis, y (*n* = 101, 52, 49)	28 (24–31)	29 (25–31)	28 (23–31)	0.795
HIV diagnosis (*n* = 114, 65, 49)				
Before conception	91 (80)	47 (72)	44 (90)	0.021
During pregnancy	22 (19)	17 (26)	5 (10)	0.033
Postpartum	1 (1)	1 (2)	0 (0)	1.000
Gestation at HIV diagnosis in patients diagnosed during pregnancy, weeks + days (*n* = 21, 17, 4)	13^+0^ (11^+0^–16^+5^)	15^+0^ (12^+0^–19^+5^)	11^+5^ (10^+4^–12^+5^)	0.140
Late HIV diagnosis (*n* = 23, 18, 5)^c^	7 (30)	5 (28)	2 (40)	0.621
Very late HIV diagnosis (*n* = 23, 18, 5)^d^	6 (26)	4 (22)	2 (40)	0.576
CDC disease stage (*n* = 113, 66, 47)				
Asymptomatic	103 (91)	59 (89)	44 (94)	0.518
Symptomatic	2 (2)	1 (2)	1 (2)	1.000
AIDS	8 (7)	6 (9)	2 (4)	0.466

RMP, regular male partner.

^a^Sample sizes for each period are given as (n = 2008–19, 2008–13, 2014–19).

^b^Values are given as number (percentage) or median (interquartile range).

^c^CD4 count < 350 cells/mm^3^.

^d^CD4 count < 200 cells/mm^3^.

^e^P-value for comparison between 2008–2013 and 2014–2019.

Thirty-five percent of pregnancies were unplanned, with a decrease from 43 to 26% across the two time periods (*p* = 0.060; Table 2). The rates of unplanned pregnancies among women diagnosed with HIV before and after conception were very similar (35%, compared to 38%), with each decreasing over time. These findings are in line with the high proportion of patients who did not use any contraception (44%), which remained unchanged between the time periods. Forty-one percent of WLHIV booked late (≥13 weeks), with a reduction from 47 to 33% between the two periods (*p* = 0.155). Of late bookers, 3 WLHIV (7%) booked during the third trimester. Eighteen percent of WLHIV were nulliparous, with a decrease over time from 21 to 14% (*p* = 0.361; Table 2).

**Table 2:** Contraception, booking, and obstetric history.

**Characteristic^a^**	**Whole cohort (2008–19)^b^**	**2008–13^b^**	**2014–19^b^**	***P*-value^c^**
Pregnancy unplanned (*n* = 110, 63, 47)	39 (35)	27 (43)	12 (26)	0.060
Among women diagnosed with HIV before pregnancy (*n* = 86, 44, 42)	30 (35)	18 (41)	12 (29)	0.230
Among women diagnosed with HIV during pregnancy (*n* = 24, 19, 5)	9 (38)	9 (47)	0 (0)	0.118
Last method(s) of contraception used (*n* = 86, 55, 31)				
Condom	24 (26)	15 (27)	9 (24)	0.861
Depo-Provera	8 (9)	6 (11)	2 (5)	0.705
Implant	4 (4)	3 (5)	1 (3)	1.000
Coil	5 (5)	3 (5)	2 (5)	1.000
Oral contraceptive pill	3 (3)	2 (4)	1 (3)	1.000
Condom and oral contraceptive pill	1 (1)	1 (2)	0 (0)	1.000
None	41 (44)	25 (45)	16 (43)	0.583
Last menstrual period known (*n* = 111, 65, 46)	88 (79)	51 (78)	37 (80)	0.801
Twin pregnancy (*n* = 116, 67, 49)	3 (3)	0 (0)	3 (6)	0.073
IVF pregnancy^d^ (*n* = 116, 67, 49)	8 (7)	2 (3)	6 (12)	0.069
Gestation at booking, weeks + days (*n* = 110, 62, 48)	12^+0^ (9^+5^–14^+6^)	12^+6^ (9^+6^–16^+0^)	11^+4^ (9^+5^–13^+4^)	0.250
Late booking at ≥13 weeks (*n* = 110, 62, 48)	45 (41)	29 (47)	16 (33)	0.155
Parity (*n* = 116, 67, 49)				
Nulliparous	21 (18)	14 (21)	7 (14)	0.361
1	32 (28)	20 (30)	12 (24)	0.532
2	34 (29)	16 (24)	18 (37)	0.133
≥3	29 (25)	17 (25)	12 (24)	0.914
History of termination of pregnancy (*n* = 116, 67, 49)	25 (22)	17 (25)	8 (16)	0.242
History of miscarriage (*n* = 116, 67, 49)	42 (36)	21 (31)	21 (43)	0.203
History of preterm birth (*n* = 109, 64, 45)	6 (6)	2 (3)	4 (9)	0.228
History of stillbirth (*n* = 116, 67, 49)	5 (4)	1 (2)	4 (8)	0.161

^a^Sample sizes for each period are given as (*n* = 2008–19, 2008–13, and 2014–19).

^b^Values are given as number (percentage) or median (interquartile range).

^c^P-value for comparison between 2008–2013 and 2014–2019.

^d^IVF, *in vitro* fertilization.

We found a significant increase in the proportion of WLHIV taking ART at conception, from 54% in 2008–2013 to 84% in 2014–2019 (*p* = 0.001; Table 3). In WLHIV taking ART at conception, 86% had a viral load of <50 copies/mL at booking, compared to only 5% of WLHIV not on ART at conception (*p* < 0.00001). The median CD4 count at booking was 560 cells/mm^3^ in WLHIV on ART at conception, compared to 350 cells/mm^3^ in those who had not commenced ART (*p* = 0.0003; Table 3). All WLHIV who were not on cART at conception commenced cART during pregnancy, except for one woman with a concealed pregnancy who was diagnosed with HIV postnatally. The median gestation at which cART was initiated decreased from 22^+1^ weeks in 2008–2013 to 17^+1^ weeks in 2014–2019 (*p* = 0.003).

**Table 3:** HIV and antiretroviral therapy characteristics.

**Characteristic^a^**	**Whole cohort (2008–19)^b^**	**2008–13^b^**	**2014–19^b^**	***P*-value^f^**
Timing of ART (*n* = 116, 67, 49)				
Taking ART at conception	77 (66)	36 (54)	41 (84)	0.001
Started ART during pregnancy	37 (32)	29 (43)	8 (16)	0.002
No ART in pregnancy	1 1 (1)	1 (1)	0 (0)	1.000
Unknown	1 (1)	1 (1)	0 (0)	1.000
Taking ART at conception (*n* = 77, 36, 41)				
CD4 count at booking, cells/mm^3^ (*n* = 77, 36, 41)	560 (420–660)	545 (360–643)	560 (430–660)	0.689
Viral load < 50 copies/mL at booking (*n* = 77, 36, 41)	66 (86)	29 (81)	37 (90)	0.225
Viral load >50 copies/mL at booking (*n* = 77, 36, 41)	11 (14)	7 (19)	4 (10)	-
Viral load at booking, copies/mL (*n* = 11, 7, 4)	237 (133–393)	237 (113–461)	253 (186–326)	1.000
Started ART during pregnancy (*n* = 37, 29, 8)				
Indication for starting ART (*n* = 34, 29, 5)				
Prevention of vertical HIV transmission	28 (82)	26 (90)	2 (40)	0.029
CD4 count < 350 cells/mm^3^	3 (9)	3 (10)	0 (0)	1.000
Universal treatment	3 (9)	0 (0)	3 (60)	0.002
Gestation when ART regimen started, weeks + days (*n* = 34, 26, 8)	22^+0^ (18^+5^–24^+5^)	22^+1^ (20^+1^–25^+0^)	17^+1^ (14^+4^–20^+1^)	0.003
CD4 count at booking, cells/mm^3^ (*n* = 37, 29, 8)	350 (230–510)	350 (230–480)	405 (248–533)	0.826
Viral load < 50 copies/mL at booking (*n* = 37, 29, 8)	2 (5)	2 (7)	0 (0)	1.000
Viral load >50 copies/mL at booking (*n* = 37, 29, 8)	35 (95)	27 (93)	8 (100)	-
Viral load at booking, copies/mL (*n* = 35, 27, 8)	14,390 (6,478–42,559)	14,390 (8,363–48,820)	12,806 (2,862–23,425)	0.317
Initial ART regimen (*n* = 115, 66, 49)				
Zidovudine monotherapy	1 (1)	1 (2)	0 (0)	1.000
PI-based cART	61 (53)	38 (58)	23 (47)	0.258
NNRTI-based cART	46 (40)	26 (39)	20 (41)	0.878
INSTI-based cART	6 (5)	0 (0)	6 (12)	0.005
PI + INSTI-based cART	1 (1)	1 (2)	0 (0)	1.000
ART regimen changed (*n* = 113, 65, 48)	20 (18)	13 (20)	7 (15)	0.456
Reason for change (*n* = 20, 13, 7)^c^				
Treatment failure	8 (36)	6 (43)	2 (25)	0.642
Intolerance	8 (36)	4 (29)	4 (50)	0.356
Toxicity	2 (9)	2 (14)	0 (0)	0.521
Other or unknown reason	4 (18)	2 (14)	2 (25)	0.587
Changes in ART regimen^d^				
Zidovudine monotherapy to PI-based cART (*n* = 1, 1, 0)	1 (100)	1 (100)	0 (0)	1.000
NNRTI-based cART → PI-based cART (*n* = 46, 26, 20)	1 (2)	1 (4)	0 (0)	1.000
PI-based cART → NNRTI-based cART (*n* = 61, 38, 23)	1 (2)	1 (3)	0 (0)	1.000
PI-based cART → INSTI-based cART (*n* = 61, 38, 23)	3 (5)	1 (3)	2 (9)	0.551
PI-based cART → PI + INSTI-based cART (*n* = 61, 38, 23)	6 (10)	3 (8)	3 (13)	0.664
Other ART changes^e^ (*n* = 115, 66, 49)	8 (7)	5 (8)	3 (6)	0.721
Final ART regimen (*n* = 113, 64, 49)				
Zidovudine monotherapy	0 (0)	0 (0)	0 (0)	1.000
PI-based cART	51 (45)	33 (52)	18 (37)	0.116
NNRTI-based cART	46 (41)	26 (41)	20 (41)	0.984
INSTI-based cART	9 (8)	1 (2)	8 (16)	0.010
PI + INSTI-based cART	7 (6)	4 (6)	3 (6)	1.000
CD4 count closest to delivery, cells/mm^3^ (*n* = 114, 66, 48)	505 (393–660)	455 (350–668)	535 (428–645)	0.435
Time of diagnosis				
Before pregnancy (*n* = 90, 47, 43)	535 (420–685)	500 (400–700)	540 (470–665)	0.594
During pregnancy or postpartum (*n* = 22, 17, 5)	445 (293–560)	450 (290–560)	340 (300–420)	0.369
Initiation of treatment				
Before pregnancy (*n* = 76, 36, 40)	535 (410–663)	530 (373–705)	535 (460–645)	0.743
During pregnancy or postpartum (*n* = 37, 29, 8)	450 (350–660)	450 (350–660)	495 (338–650)	0.835
Viral load < 50 copies/mL closest to delivery (*n* = 116, 67, 49)	104 (90)	58 (87)	46 (94)	0.235
Time of diagnosis				
Before pregnancy (*n* = 91, 47, 46)	82 (90)	41 (87)	41 (89)	0.777
During pregnancy (*n* = 23, 18, 5)	20 (87)	15 (83)	5 (100)	1.000
Initiation of treatment				
Before pregnancy (*n* = 77, 36, 41)	70 (91)	32 (89)	38 (93)	0.699
During pregnancy (*n* = 37, 29, 8)	33 (89)	25 (86)	8 (100)	0.557
Viral load ≥50 copies/mL closest to delivery (*n* = 116, 67, 49)	12 (10)	9 (13)	3 (6)	0.235
Viral load closest to delivery, copies/mL (*n* = 12, 9, 3)	114 (52–1,100)	100 (52–3,140)	220 (135–1,063)	0.840

ART, antiretroviral therapy; cART, combination antiretroviral therapy (triple drug therapy); PI, protease inhibitor; NRTI, nucleoside reverse transcriptase inhibitor; NNRTI, non-nucleoside reverse transcriptase inhibitor; INSTI, integrase strand transfer inhibitor.

^a^Sample sizes for each period are given as (*n* = 2008–19, 2008–13, and 2014–19).

^b^Values are given as number (percentage) or median (interquartile range).

^c^Two patients switched treatment twice, hence the total number of reasons for treatment change is greater than the number of patients who changed.

^d^Values for “*n*” indicate number of patients who started treatment on the specified regimen.

^e^Including stopping, adding, or switching drugs with no change in the regimen type.

^f^*P*-value for comparison between 2008–2013 and 2014–2019.

**Figure 1:**
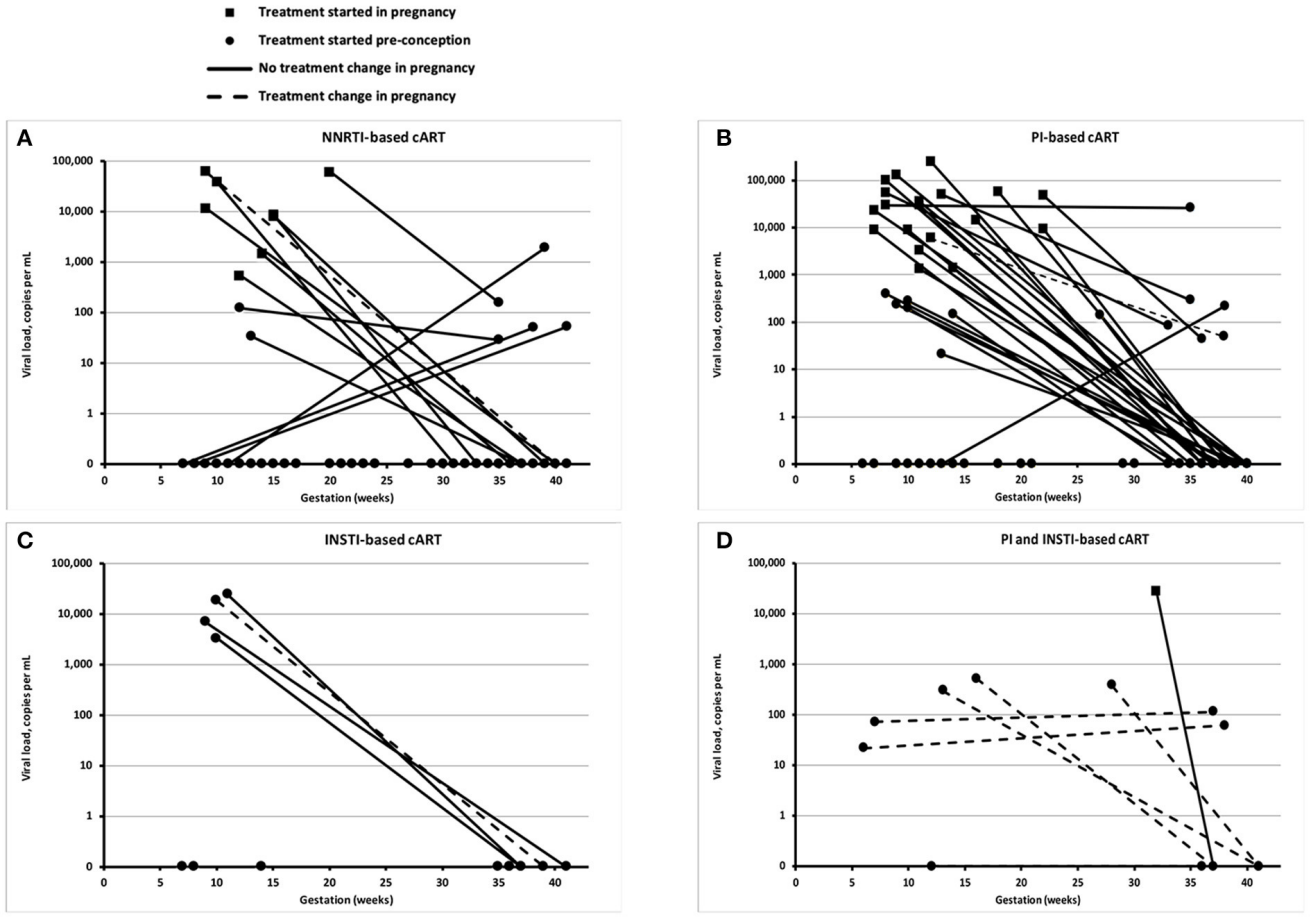
Viral loads at booking and delivery. Each panel shows the viral load of individual women living with HIV (WLHIV) at booking and delivery, according to the type of combination antiretroviral therapy (cART) at delivery: NNRTI-based cART **(A)**, PI-based cART **(B)**, INSTI-based cART **(C)**, and PI and INST-based cART **(D)**. Note that some detectable viral loads at delivery are <50 copies/mL, including one WLHIV on NNRTI-based cART and two WLHIV on PI-based cART.

Most WLHIV received either protease inhibitor (PI)-based cART, or non-nucleoside reverse transcriptase inhibitor (NNRTI)-based cART (Table 3, Figure 1). Between the two time periods, the proportion of WLHIV on integrase inhibitor (INSTI)-based cART at delivery rose from 2 to 16% (*p* = 0.010). Overall, 18% of WLHIV had a change in their cART regimen during pregnancy, most commonly either adding an INSTI or switching to an INSTI-based regimen.

At delivery, 90% of all WLHIV had a viral load <50 copies/mL (Table 3). The proportion of WLHIV with a viral load <50 copies/mL at delivery was similar regardless of the timing of diagnosis or treatment initiation (Table 3). Conversely, preconception diagnosis was associated with a statistically significant higher CD4 count at delivery (median 535 vs. 445 cells/mm^3^; *p* = 0.029).

Amongst the 12 WLHIV with a viral load of ≥50 copies/mL at delivery, eight had a viral load between 50 and 500 copies/mL, and four >10,000 copies/mL. Of this cohort, nine were diagnosed with HIV preconception, and seven were on preconception cART. Of the five women not on preconception cART, four started cART during pregnancy, and one postnatally. Most of the 12 women with a viral load of ≥50 copies/mL at delivery started pregnancy with very high viral loads, with a median booking viral load of 52,141 copies/mL. This compared to a median viral load of 8,106 copies/mL in WLHIV who also had a viral load ≥50 copies/mL at booking, but a viral load <50 copies/mL at delivery (*p* = 0.086). Two WLHIV who had a viral load ≥50 copies/mL at delivery self-reported poor adherence to ART. Four WLHIV changed ART regimen during pregnancy, including three due to their persistently high viral loads, and one due to treatment side effects. By delivery, four of the 12 WLHIV received NNRTI-cART, four PI-cART, and two PI-/INSTI-cART, with cART regimen unknown for one WLHIV and no cART received by the one WLHIV with a concealed pregnancy.

Overall, 34% of WLHIV delivered vaginally and 66% by Cesarean section (Table 4). There was a significant increase in the number of WLHIV planning for a vaginal delivery from 38 to 61% (*p* = 0.016), which led to an increase in vaginal deliveries from 27 to 43% (*p* = 0.072), including a significant increase in forceps deliveries from 1 to 12% (*p* = 0.041; Table 4). There was a significant reduction in the proportion of WLHIV planning an elective Cesarean section from 62 to 39% (*p* = 0.016), which translated into a significant reduction in actual deliveries by elective Cesarean section from 49 to 31% (*p* = 0.044).

**Table 4:** Delivery and neonatal characteristics.

**Characteristic^a^**	**Whole cohort (2008–19)^b^**	**2008–13^b^**	**2014–19^b^**	***P*-value^e^**
Planned mode of delivery (*n* = 114, 65, 49)				
Elective cesarean	59 (52)	40 (62)	19 (39)	0.016
Vaginal delivery	55 (48)	25 (38)	30 (61)	-
Actual mode of delivery (*n* = 116, 67, 49)				
Cesarean section	77 (66)	49 (73)	28 (57)	0.072
Elective cesarean	48 (41)	33 (49)	15 (31)	0.044
Emergency cesarean	29 (25)	16 (24)	13 (27)	0.745
Vaginal delivery	39 (34)	18 (27)	21 (43)	0.072
Normal vaginal delivery	32 (28)	17 (25)	15 (31)	0.533
Forceps delivery	7 (6)	1 (1)	6 (12)	0.041
Other intrapartum interventions (*n* = 49)				
Continuous electronic fetal monitoring	-	-	12 (24)	-
Induction of labor	-	-	12 (24)	-
Artificial rupture of membranes	-	-	14 (29)	-
Oxytocin	-	-	15 (31)	-
Fetal blood sampling	-	-	0 (0)	-
Fetal scalp electrode	-	-	0 (0)	-
Episiotomy	-	-	5 (10)	-
Instrumental delivery	-	-	6 (12)	-
Mode of delivery influenced by inability to perform intrapartum interventions due to HIV status (*n* = 49)				
Yes	-	-	2 (4)	-
No	-	-	47 (96)	-
Intrapartum zidovudine (*n* = 111, 63, 48)	47 (42)	45 (71)	2 (4)	< 0.0001
Delivery outcome (*n* = 119, 67, 52)				
Live birth	117 (98)	66 (99)	51 (98)	1.000
Stillborn	2 (2)	1 (1)	1 (2)	-
Gestation at delivery (*n* = 116, 67, 49)				
< 32 weeks	3 (3)	2 (3)	1 (2)	1.000
32-37 weeks	10 (9)	4 (6)	6 (12)	0.319
>37 weeks	103 (89)	61 (91)	42 (86)	0.369
Birth weight, kg (*n* = 119, 67, 52)	3.05 (0.62)	3.04 (0.55)	3.08 (0.70)	0.722
>3.0 kg	66 (55)	36 (54)	30 (58)	0.666
2.5-3.0 kg	34 (29)	22 (33)	12 (23)	0.242
< 2.5 kg	19 (16)	9 (13)	10 (19)	0.392
Gestation adjusted weight (*n* = 119, 67, 52)				
SGA^c^	18 (15)	13 (19)	5 (10)	0.139
AGA	91 (76)	50 (75)	41 (79)	0.590
LGA^d^	10 (8)	4 (6)	6 (12)	0.330
ART in neonate (*n* = 103, 53, 50)				
Zidovudine monotherapy	94 (91)	45 (85)	49 (98)	0.019
cART	4 (4)	4 (8)	0 (0)	0.118
cART followed by zidovudine	3 (3)	2 (4)	1 (2)	1.000
None	2 (2)	2 (4)	0 (0)	0.496
Congenital abnormalities (*n* = 110, 63, 47)	7 (6)	4 (6)	3 (6)	0.994
Neonate HIV positive (*n* = 114, 66, 48)	1 (1)	1 (2)	0 (0)	1.000

SGA, small for gestational age (< 10th centile); AGA, appropriate for gestational age (10–90th centile); LGA, large for gestational age (>90th centile); cART, combination antiretroviral therapy.

^a^Sample sizes for each period are given as (*n* = 2008–19, 2008–13, and 2014–19).

^b^Values are given as number (percentage) or mean (standard deviation).

^c^ < 10th centile.

^d^>90th centile.

^e^*P*-value for comparison between 2008–2013 and 2014–2019.

Key findings in relation to intrapartum interventions included a significant decrease in the proportion of WLHIV treated with intrapartum zidovudine, from 71 to 4% (*p* = < 0.0001; Table 4). In only two patients in the period 2014–2019 was the mode of delivery influenced by a choice not to perform an intrapartum intervention (fetal blood sampling and fetal scalp electrode) due to the woman's HIV status.

In total, 119 babies were born to 116 WLHIV, including three sets of twins and two stillbirths. One stillbirth occurred at 38 weeks secondary to a placental abruption. The other occurred at 31 weeks, with unknown cause. Overall, there were 13 (11%) preterm births (<37 weeks), including three very preterm births (<32 weeks) and 15% of infants were small for gestational age (SGA), without significant changes between the two periods (Table 4).

Across the two studied time periods, the proportion of infants treated with zidovudine increased from 85 to 98% (*p* = 0.019; Table 4). There was a corresponding fall in the proportion of infants treated with cART from 12 to 2%. Amongst the 12 infants born to WLHIV with viral loads ≥50 copies/mL at delivery, three were treated with zidovudine alone (the highest maternal viral load in this group was 220), two were treated with cART alone, two received a regimen of cART followed by zidovudine, one infant received neither zidovudine nor cART, and in four the regimen was unknown.

One baby tested positive for HIV (0.86%), which occurred in the period 2008–2013. The woman was diagnosed with HIV and ART initiated preconception, but her viral load control was poor throughout pregnancy. At booking at 21 weeks, she had a viral load of >60,000 copies/mL. Her treatment was changed during pregnancy due to treatment failure, but her adherence was reported to be poor. Although she planned for a Cesarean section, she delivered vaginally at 32 weeks, when her viral load was >30,000 copies/mL. Cesarean section and treatment with intrapartum zidovudine were not possible due to a rapid delivery.

Timing of HIV diagnosis, booking, or ART initiation were not associated with adverse outcomes (Table 5). Protease inhibitors, viral load at booking and CD4 count were also not associated with perinatal outcomes. In contrast, a viral load of ≥50 copies/mL at delivery was associated with preterm birth, with 42% of WLHIV with a viral load ≥50 copies/mL delivering preterm, compared to 8% of WLHIV with a viral load of <50 copies/mL at delivery (*p* = 0.0004; Table 5). The preterm births among the WLHIV with a viral load of ≥50 copies/mL included three moderate to late preterm births (32–37 weeks), one very preterm birth (28–32 weeks), and one extreme preterm birth (< 28 weeks).

**Table 5:** Associations between HIV and treatment characteristics, and birth outcomes.

**Characteristic^a^**	**Preterm births, *n* (%)^b^**	**Median birth weight, kg^c^**	**Birth weight adjusted for gestational age** ^**c**^		
			**SGA**	**AGA**	**LGA**
Time of HIV diagnosis					
Before pregnancy (*n* = 91, 94, 94)	10 (11)	3.11	11	76	7
During pregnancy (*n* = 23, 23, 23)	2 (9)	2.86	7	13	3
*P*-value	1.000	0.286	0.228		
Time of booking					
Early (< 13 weeks; *n* = 65, 65, 65)	5 (8)	3.10	10	49	6
Late (≥13 weeks; *n* = 45, 48, 48)	6 (13)	3.13	8	38	2
*P*-value	0.332	0.736	0.622		
Protease inhibitor treatment^d^					
Yes (*n* = 64, 66, 66)	6 (9)	3.02	12	51	3
No (*n* = 52, 53, 53)	7 (13)	3.16	6	40	7
*P*-value	0.488	0.078	0.166		
Initiation of treatment					
Pre-conception (*n* = 77, 80, 80)	8 (10)	3.12	11	62	7
Post-conception (*n* = 38, 38, 38)	5 (13)	2.95	7	28	3
*P*-value	0.659	0.392	0.790		
Viral load at booking, copies/mL					
< 50 (*n* = 68, 70, 70)	8 (12)	3.14	9	54	7
≥50 (*n* = 48, 49, 49)	5 (10)	2.92	9	37	3
*P*-value	0.821	0.153	0.611		
Viral load at delivery, copies/mL					
< 50 (*n* = 104, 107, 107)	8 (8)	3.10	18	80	9
≥50 (*n* = 12, 12, 12)	5 (42)	2.96	0	11	1
*P*-value	0.0004	0.093	0.289		
CD4 count at booking, cells/mm^3^					
≥350 (*n* = 85, 87, 87)	8 (9)	3.10	13	68	6
< 350 (*n* = 29, 30, 30)	4 (14)	3.03	5	22	3
*P*-value	0.498	0.718	0.806		
CD4 count at delivery, cells/mm^3^					
≥350 (*n* = 92, 94, 94)	9 (10)	3.10	14	72	8
< 350 (*n* = 23, 24, 24)	4 (17)	3.03	4	18	2
*P*-value	0.290	0.410	0.924		

SGA, small for gestational age (< 10th centile); AGA, appropriate for gestational age (10–90th centile); LGA, large for gestational age (>90th centile).

^a^Sample sizes for perinatal outcome analyzed are given as [n = number of pregnancies (preterm birth analysis), number of infants (birth weight analysis), and number of infants (birth weight adjusted for gestational age analysis)].

^b^Values correspond to pregnancies.

^c^Values correspond to infants.

^d^Women in the “yes” group include all women exposed to a PI at any time in pregnancy, regardless of the duration of PI treatment.

## Discussion

This study identified a number of improvements in the management and outcomes among WLHIV delivering at a large tertiary obstetric unit in the UK during 2008–2019. WLHIV delivering in more recent years were more likely to be diagnosed with HIV before conception, to be on ART at conception, and, when ART was started in pregnancy, it was started at an earlier gestational age. The proportion of WLHIV receiving integrase inhibitors increased and the proportion of WLHIV with a viral load ≥50 copies/mL at delivery reduced over time. WLHIV were less likely to both plan for and deliver by elective Cesarean. There were no perinatal HIV transmission during 2014–2019 and other perinatal outcomes remained unchanged.

The rate of late booking in this study was lower than the national average for WLHIV with HIV (41%, compared to 51%) (23). However, it remains considerably higher than a previously reported national average of 15% across all women (24). Further work is required to understand the factors causing late booking in this population, particularly as late booking has been identified as a major contributory factor in cases of vertical HIV transmission in the UK (25).

The rate of antenatal HIV diagnosis in this cohort (19%) is comparable to other data from both the UK and cohorts in other high-income countries (8, 26–30). In keeping with a national decline in new antenatal diagnoses, the proportion of women diagnosed with HIV in pregnancy in our cohort fell from 27% in 2008–2013 to 10% in 2014–2019. This was reflected by the high proportion of WLHIV (66%) already on treatment at conception, which rose significantly from 54 to 84%. In comparison, rates of ART use at conception were lower both nationally and at two other centers reporting recently, ranging from 49 to 76% (6, 8, 26, 31, 32). In WLHIV who started ART in pregnancy, the median gestation at ART initiation fell from 22 to 17 weeks. This reflects similar progress in other national and international cohorts, with recent studies reporting a median start time of 22 weeks (6, 31, 33) compared to 24–28 weeks in earlier studies (33–35).

We found an increase in the proportion of WLHIV receiving an integrase inhibitor during pregnancy, reaching 22% at delivery during 2014–2019. A recent randomized controlled trial (RCT) reported that integrase inhibitor dolutegravir has superior virological efficacy at delivery and may improve perinatal outcomes, compared to efavirenz-based cART, when initiated during pregnancy (36). The WHO and US guidelines recommend dolutegravir-based cART as first-line regimen, but the current BHIVA guideline recommends integrase inhibitors as second-line drugs (37–39). It is anticipated that the UK guideline will soon be updated and recommend dolutegravir-based cART as first-line for use in pregnant and breastfeeding WLHIV, which should lead to an increase in its use in pregnancy.

The proportion of WLHIV with a viral load <50 copies/mL at delivery (90% overall, and 94% in the most recent period) is higher than in other studies from the last 10 years (6, 28, 29, 31, 33, 35, 40), and higher than the national figure of 87% between 2012 and 2014 (8). The proportion of WLHIV delivering vaginally in the present study increased from 27 to 43%. This is similar to recent national data from 2012 to 2014, which reported a vaginal delivery rate of 46% (8). This increase in the rate of vaginal delivery is likely due to guidance encouraging vaginal delivery for WLHIV with a viral load <50 copies/mL, even when the duration of membrane rupture has been >4 h (3). Forceps, as opposed to Ventouse, is the instrument of choice for assisted vaginal deliveries in the context of HIV and its use increased from 1 to 12%, in line with instrumental rates in pregnancies not affected by HIV.

Vertical transmission of HIV occurred in one delivery (0.86%). This is similar to the national finding of a 0.27% transmission rate between 2012 and 2014 (8), as well as rates reported from both the UK and other high-income countries over the last 10 years (26, 28, 30, 31, 33, 35, 41, 42). The preterm delivery rate of 11.2% is lower than the national rate of 12.5% amongst WLHIV, but remains considerably higher than the national rate of 7.8% amongst all women (14, 43). The rate of low birth weight was 16%. This is comparable to national UK data (44), whilst other similar cohorts in high-income countries report rates ranging from 12 to 25% (28, 41, 42, 45, 46). Fifteen percent of infants were SGA. In comparison, other studies in WLHIV reported rates of SGA birth ranging from 7 to 26%, whilst the national rate of SGA birth is 9% (15–19). Two infants (2%) in the present study were stillborn. This compares to a national rate of 0.85% between 2007 and 2015 (20). We did not find an association between preterm birth or birth weight and late booking, antenatal diagnosis, late treatment initiation, use of protease inhibitors, high viral load at booking, or low CD4 count. The finding of an association between a viral load of ≥50 copies/mL and preterm birth highlights the importance of early initiation of ART during pregnancy and achieving virological control early in pregnancy. The increased use of dolutegravir should facilitate rapid suppression of viral load during pregnancy.

Our detailed analyses show that over time more WLHIV are diagnosed before pregnancy and more WLHIV initiate ART before pregnancy, leading to higher levels of viral suppression (and higher CD4 counts) at delivery, which in turn leads to more vaginal births and fewer cases of perinatal HIV transmission. We believe this demonstrates that implementation of regularly updated national HIV guidelines is instrumental in improving pregnancy outcomes for WLHIV (3). Integration of HIV care into obstetric and neonatal care pathways is key to achieving good outcomes. This was achieved through a multidisciplinary approach involving obstetricians, infectious disease/HIV physicians, support workers and neonatologists. Our data indicate the importance of preconception HIV diagnosis and ART initiation in achieving good virological control at delivery and this should be a priority in both high and low resource settings.

A major strength of our study is the comprehensive information collected over a long period, allowing a detailed analysis of changes in management and outcomes over time. The main limitation of our study is its retrospective study design, resulting in incomplete data for some variables and a risk of bias. In addition, the sample size of WLHIV delivering with a viral load ≥50 copies/mL at delivery was low, limiting our ability to draw conclusions about contributing factors to these cases. Furthermore, our statistical analysis focuses on whether several key treatment and virological characteristics are associated with perinatal outcomes. However, it is worth noting that there are other possible confounding factors that were not considered in this study.

Virological, obstetric and perinatal outcomes and trends in our cohort of pregnant WLHIV are largely favorable. However, there is room for further improvement, in particular regarding the further normalization of the management of labor for WLHIV with a viral load <50 copies/mL, promotion of vaginal delivery, and support of breastfeeding for WLHIV who opt to do so (3, 47). Further improvements in virological control and perinatal outcomes are anticipated with the increased use of integrase inhibitor dolutegravir.

## Data availability statement

The original contributions presented in the study are included in the article/supplementary material, further inquiries can be directed to the corresponding author/s.

## Ethics statement

The studies involving human participants were reviewed and approved by Oxford University Hospitals National Health Service Foundation Trust Clinical Governance Office, Level 4 Women's Center, John Radcliffe Hospital Oxford OX3 9DU. Written informed consent for participation was not required for this study in accordance with the national legislation and the institutional requirements.

## Author contributions

OB identified participants, obtained medical records, extracted data, conducted the analyses, interpreted the data, and wrote the first draft of the manuscript. SC identified participants, obtained medical records, and edited the manuscript. JH conceived, designed, and coordinated the study, developed the data collection proforma, assisted with data extraction, interpreted the data, and wrote the manuscript. All authors read and approved the final version of the manuscript.

## Conflict of interest

The authors declare that the research was conducted in the absence of any commercial or financial relationships that could be construed as a potential conflict of interest.

## Publisher's note

All claims expressed in this article are solely those of the authors and do not necessarily represent those of their affiliated organizations, or those of the publisher, the editors and the reviewers. Any product that may be evaluated in this article, or claim that may be made by its manufacturer, is not guaranteed or endorsed by the publisher.
